# Genome-wide characterization and expression analysis of the **a**uxin response factor (ARF) gene family during melon (*Cucumis melo* L.) fruit development

**DOI:** 10.1007/s00709-020-01484-2

**Published:** 2020-02-11

**Authors:** Bei Wu, Lu Wang, Gaoyang Pan, Ting Li, Xin Li, Jinghong Hao

**Affiliations:** 1 Beijing Key Laboratory for Agricultural Application and New Technology, National Demonstration Center for Experimental Plant Production Education, College of Plant Science and Technology, Beijing University of Agriculture, Beijing, 102206 China; 2 Beijing Agricultural Technology Extension Station, Beijing, 100029 China; 3 Agricultural and Rural Bureau of Jing County of Hebei Province, Hebei, 053500 China

**Keywords:** Melon, Fruit development, Auxin response factors, Expression, qRT-PCR

## Abstract

ARFs in plants mediate auxin signaling transduction and regulate growth process. To determine genome-wide characterization of ARFs family in melon (*Cucumis melo* L.), ARFs were identified via analysis of information within the melon genomic database, and bioinformatic analyses were performed using various types of software. Based on different treatment methods involving dipping with the growth regulator Fengchanji No. 2 and artificial pollination, Jingmi No. 11 melon was used as the test material, and melon plants with unpollinated ovaries served as controls. The expression of *ARF*s during the early development of melon was analyzed via qRT-PCR. Seventeen genes that encode ARF proteins were identified in the melon genome for the first time. The expression of these *ARF*s differed in different tissues. The expression levels of *CmARF2*, *CmARF16-like*, *CmARF18-like2*, and *CmARF19-like* were especially high in melon fruits. The expression of *ARF*s during the early development of melon fruits differed in response to the different treatments, which suggested that *CmARF9*, *CmARF16-like*, *CmARF19-like*, *CmARF19*, *CmARF1*, *CmARF2*, *CmARF3*, and *CmARF5* may be associated with melon fruit growth during early development. Interestingly, the increase in the transverse diameter of fruits treated with growth regulators was significantly greater than that of fruits resulting from artificial pollination, while the increase in the longitudinal diameter of the fruits resulting from artificial pollination was significantly greater.

## Introduction

Melon (*Cucumis melo* L.) is an important horticultural crop species that is cultivated in temperate, subtropical, and tropical regions worldwide. Auxin is a plant hormone that plays pivotal roles in the regulation of plant growth in response to various developmental and environmental events, such as embryogenesis, organogenesis, trophic growth, root architecture development, flower and fruit development, tissue and organ patterning, and vascular development (Davies 1987; Zhang et al. 2012; Vanneste and Friml 2009). It has been shown that auxin coordinates plant development essentially via the transcriptional regulation of members of certain gene families, such as the auxin/indole-3-acetic acid (Aux/IAA), Gretchen Hagen 3 (GH3), small auxin-up RNA (SAUR), and auxin response factor (ARF) families (Tiwari et al. 2001; Park et al. 2007; ElsharkawyI et al. 2014). Recent studies have shown that auxin signal transduction may be accomplished via the regulation of ARFs. These so-called early auxin-responsive genes are characterized by conserved promoter elements, such as the TGA element (AACGAC), core element of the auxin response region (AuxRE-core; GGTCCAT), and auxin response element (AuxRE; TGTCTC) (Guilfoyle and Hagen 2007; Tiwari et al. 2003). As important components of the auxin signaling pathway, ARFs activate or repress the expression of auxin-responsive genes by binding to AuxREs in the promoters of those genes (Ulmasov et al. 1999).

A typical ARF consists of a highly conserved N-terminal B3-like DNA-binding domain (DBD) that recognizes AuxREs in the promoters of auxin-responsive genes and a C-terminal dimerization domain (CTD) that contains two motifs, namely, III and IV, which are also found in Aux/IAA proteins and enable the formation of homodimers and heterodimers between ARFs and Aux/IAAs (Ulmasov et al. 1999). The middle region (MR), which is located between the DBD and CTD, can perform transcriptional activation or repression depending on the amino acid composition (Ellis et al. 2005). Analysis of the amino acid compositions of activation dimers (ADs) and repression dimers (RDs) of ARFs revealed that ADs are rich in glutamine (Q), serine (S), and leucine (L) residues, while RDs are rich in proline (P), serine (S), threonine (T), and glycine (G) residues (Guilfoyle and Hagen 2007; Tiwari et al. 2003; Ulmasov et al. 1999; Ellis et al. 2005).

ARF proteins participate in the transcriptional regulation of a variety of biological processes associated with growth and development, such as embryogenesis (Thorsten et al. 2002; Dolf et al. 2014), leaf expansion (Wilmoth et al. 2010), leaf senescence (Lim et al. 2010), lateral root growth (Tatematsu et al. 2004; Yoko et al. 2007), and fruit development (Marc et al. 2006; Goetz et al. 2007; et al. 2010). The genome sequencing project for melon was recently completed (Garcia-Mas et al. 2012), and the ARFs of *Arabidopsis* (Piya et al. 2014), *Oryza* (Wang et al. 2007), *Populus* (Yang et al. 2014), *Medicago* (Shen et al. 2015), and maize (Liu et al. 2011) have also been published. However, genome-wide information concerning the *ARF* gene family and the functions of ARFs in melon are largely unknown. Therefore, to improve any fruit-related agronomic traits in melon, it is necessary to study the characteristics of members of the ARF family and their potential functions during early fruit development in melon.

To elucidate the structures of CmARFs and characterize the expression of CmARFs during fruit development in melon, 17 putative genes that contain ARF domains were identified via genomic data mining. The sequence homology of all the CmARFs was then investigated, followed by a comparative phylogenetic analysis. The different temporal and spatial expression patterns during early fruit development of melon were subsequently determined for each *CmARF* gene by quantitative real-time PCR (qRT-PCR). The resulting classification of groups and characterization of the expression patterns will be useful for future analyses of the biological functions of ARF family genes in melon.

## Materials and methods

### Plant material and treatments

Seeds of melon (*Cucumis melo* L*.*) cultivar Jingmi No. 11, a popular oriental melon variety provided by the Beijing Agricultural Technology Promotion Station, were sown in an organic matter:soil (2:1 *v*/*v*) mixture at Beijing the University of Agriculture Experimental Station. The plants were cultivated under glasshouse conditions (12 h of light [300~1300 μmol m^−2^ s^−1^ light] and 26 ± 2 °C during the day; 12 h of darkness and 15 ± 2 °C during the night; 50~70% relative humidity) and were watered and fertilized in accordance with normal cultivation management practices. Female flowers were artificially pollinated or were subjected to dipping with the growth regulator Fengchanji No. 2 (a hormone complex that contains mainly 4-chlorophenoxyacetic acid to increase the rate of fruit set; Shenyang Agricultural University), and plants with unpollinated ovaries served as controls. Female flowers before 1 day of treatment were bagged to avoid pollination. The first day of blooming was considered day 0, and those plants were tagged. Plants were trained as single stems, and the fruit load was limited to three per lateral vine per plant from the 12th to 14th nodes.

Fruits were collected at 0, 1, 2, 3, and 5 days after treatment. Some of the fruit samples were used to analyze growth, and the transverse diameters and longitudinal diameters (in cm) of the fruits from the control and treated plants were measured with a Vernier caliper. The other collected fruit samples from the control and treated plants were frozen in liquid nitrogen and stored at − 80 °C until further gene expression analysis. Moreover, leaf, pistillate flower, staminate flower, stem, and root tissues from the control and treated plants were also collected for tissue-specific expression analysis.

### Identification of melon *ARF* genes

To find previously identified and potential ARF family genes in melon, multiple database searches were performed. The key term “auxin response factor” was used to query the melon database (http://melonomics.net). After searching for ARF genes, we also queried the database via the amino acid sequences of the ARF domains of several known ARF family members (including AtARFs and OsARFs). To confirm the accuracy of the gene identification further, the predicted *CmARF-like* gene sequences were compared with the sequences of ARF proteins from other species via BLASTp retrieval. Only those sequences with high scores (> 200) were selected.

### Sequence analysis

ProtParam (Mcphie 2002) (http://web.expasy.org/protparam) was used to analyze the protein parameters. The neighbor-joining (NJ) algorithm of the MEGA 6.0 program (Tamura et al. 2011) was used to construct a phylogenetic tree with Poisson correction and pairwise deletion parameters, and all other parameters were configured to the default values. A total of 1000 bootstrap replicates were included. The subcellular localization of the deduced polypeptides was predicted by WoLF PSORT (https://wolfpsort.hgc.jp/). Moreover, GSDS (Guo et al. 2007) (http:/gsds.cbi.pku.edu.cn/) was used to map the exon-intron structure of the ARF family members, and the conserved domains of the proteins were predicted using MEME Suite (http://meme-suite.org/tools/meme) (Bailey et al. 2009).

### RNA isolation and cDNA synthesis

Total RNA was isolated using TransZol (TransGen Biotech, Beijing, China) according to the manufacturer’s instructions. The RNA integrity was determined by electrophoresis on 1% agarose gels. The quantity and purity of the RNA were determined using a NanoDrop™ 2000 spectrophotometer (Thermo Fisher Scientific, Massachusetts, United States). Only high-quality samples in which A260:A280 ≥ 1.8 and A260:/A230 ≥ 2.0 were used for subsequent cDNA synthesis. cDNA was synthesized using a Prime-Script RT Reagent Kit (Perfect Real Time, TaKaRa, Tokyo, Japan) according to the manufacturer’s instructions. For each sample, 1 mg of total RNA was used for each 20-mL reverse transcription reaction system.

### Semiquantitative PCR and qRT-PCR

For semiquantitative PCR and qRT-PCR, gene-specific oligonucleotide primers for each ARF gene were designed by Beacon Designer; these primers are listed in Supplemental Table 1. The specificity of each pair of primers was determined by agarose gel electrophoresis and by resequencing the PCR products. Twenty cycles were carried out for the CmARFs.

The reaction system (10 μL) contained 2 × SYBR qPCR mix (5 μL), cDNA template (1 μL), 10 μM forward primer (0.5 μL), 10 μM reverse primer (0.5 μL), and ddH_2_O (3 μL). The reaction procedure was as follows: 3 min of predenaturation at 95 °C; 40 cycles of 10 s at 94 °C, 30 s of annealing (the annealing temperature was set according to the information in Supplemental Table 1), 20 s at 72 °C, and 5 min of extension at 72 °C (Weng et al. 2017). The 2^−ΔΔCT^ relative quantitation method was then used to calculate the relative expression of the *ARF* genes (Livak and Schmittgen 2001). The 18S rRNA gene (148 bp) of melon was used as an internal control. A Bio-Rad CFX 96 real-time PCR instrument and CFX manager 3.0 (Bio-Rad Lab., CA, USA) were used for real-time PCR analysis. Each experiment was repeated at least three times.

### Statistical analysis

The experiment was performed in triplicate. With respect to the measurements of the transverse diameters and longitudinal diameters (cm) of the fruits, each biological replicate consisted of five samples from five individual plants. With respect to the gene expression analyses, three different fruits were pooled together as one biological sample, and this was performed three times to produce three independent biological replicates (each consisting of three pooled fruits). The data represent the means ± SDs of three replications and were statistically analyzed using analysis of variance (ANOVA) by SPSS 10.0 (International Business Machine, Chicago, IL, USA). Tukey’s test was subsequently used to determine significant differences among the groups (*p* < 0.05, *p* < 0.01). Figures representing the morphological and gene expression parameters were drawn using Origin Pro 8.0 SR4 (Origin Lab, Northampton, MA, USA).

## Results

### Identification and bioinformatic analyses of melon *ARF* genes

The sequences of all the predicted ARF genes in the melon genome were compiled and compared with those of ARF genes from other species. Seventeen ARF genes with complete structural domains were ultimately identified in melon. The *CmARF*s were named on the basis of the results of the comparison with the other species. Three of the *ARF18-like* genes were distinguished by adding one number to the end.

Using ExPASy, we found that the longest ARF protein consists of 1110 amino acid residues, while the shortest ARF protein consists of 584 amino acid residues. The ORF length ranges from 1752 to 3330 bp. In addition, the isoelectric points of the 17 ARF proteins were also predicted to range from 5.42 to 8.67, as shown in Table 1. Via the WoLF PSORT program, 16 ARF proteins were determined to be located in the nucleus, whereas *CmARF5* was predicted to be located in the peroxisome.

**Table 1:** The information of ARF genes in melon

Gene	Gene accession no.	Proteinaccession no.	ORF length (bp)	Amino acid (aa)	Molecular weight (kD)	Isoelectric point
*CmARF1*	LOC103499376	XP_016902572.1	2025	675	75.11	6.02
*CmARF2*	LOC103502275	XP_008464366.1	2520	840	93.18	6.11
*CmARF3*	LOC103484487	XP_016899371.1	2199	733	79.94	6.61
*CmARF4*	LOC103501935	XP_008463923.1	2436	812	89.75	6.52
*CmARF5*	LOC103503893	XP_008466501.1	2847	949	104.42	5.42
*CmARF6*	LOC103484339	XP_008439586.1	2745	915	101.58	6.19
*CmARF6-like*	LOC103487981	XP_008444734.1	2706	902	99.88	5.95
*CmARF8*	LOC103501352	XP_008463127.1	2556	852	94.65	5.82
*CmARF9*	LOC103482831	XP_008437407.1	2082	691	77.01	5.99
*CmARF16-like*	LOC103496426	XP_008456485.1	2118	706	77.95	6.71
*CmARF17*	LOC103488417	XP_008445361.2	1752	584	60.06	6.65
*CmARF18*	LOC103501953	XP_008463952.1	2148	716	79.03	8.67
*CmARF18-like1*	LOC103491181	XP_008449245.1	2094	698	76.73	6.43
*CmARF18-like2*	LOC103493078	XP_008451925.1	2097	699	78.17	6.90
*CmARF18-like3*	LOC103500545	XP_016902809.1	1950	650	73.09	6.31
*CmARF19*	LOC103498967	XP_008460034.1	3330	1110	123.54	6.15
*CmARF19-like*	LOC103492260	XP_008450783.1	3318	1106	121.63	6.07

**Fig. 1:**
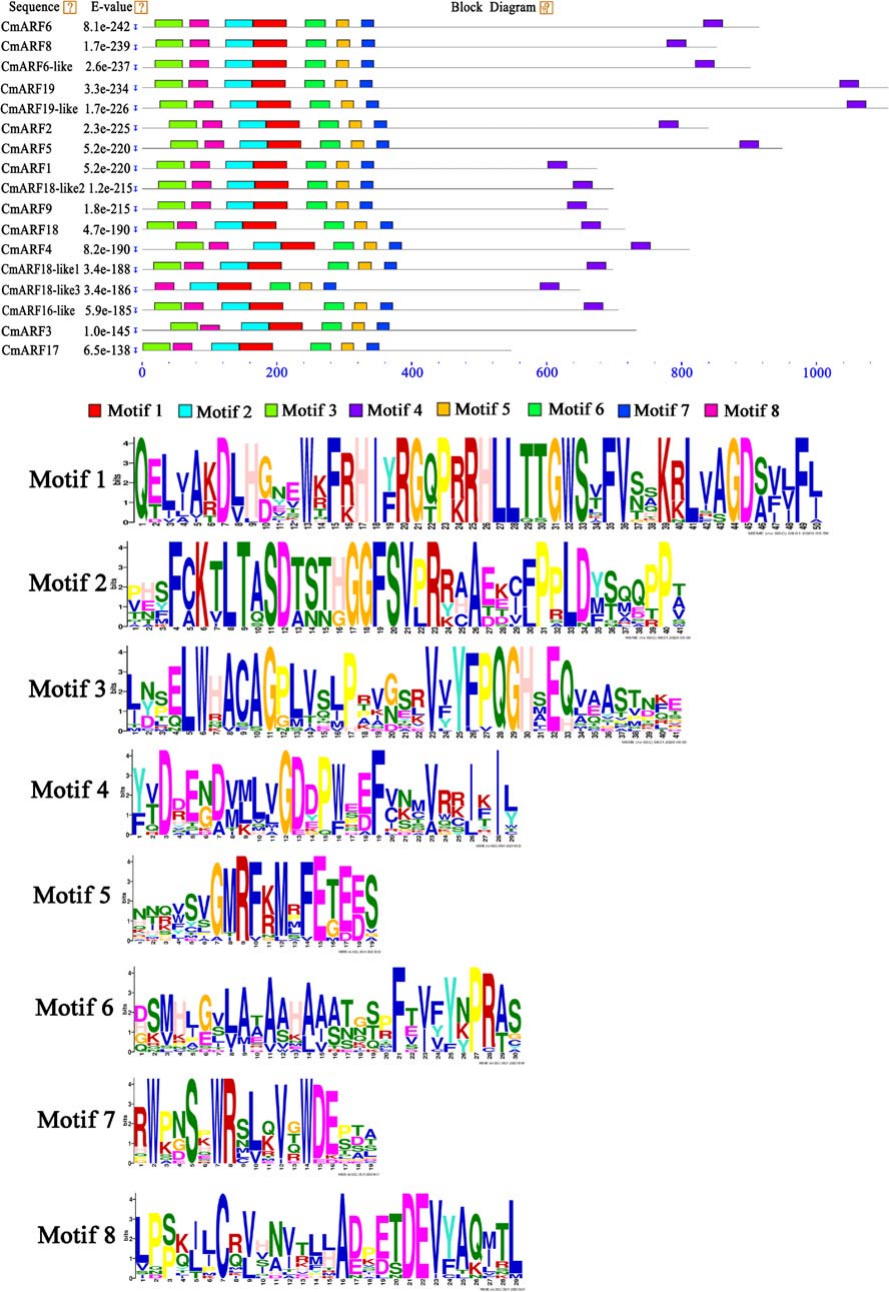
The information on ARF members in melon. The amino acid sequences of the CmARFs were analyzed by MEME software for the selection of 8 conserved sequences

The amino acid sequences of the CmARFs were analyzed by MEME software for the selection of 8 conserved sequences, as shown in Fig. 1. Motifs 1 and 2 were located in the structural DBD of the CmARFs, and motifs 5, 6, and 7 were located in the MR conserved structural domain of the CmARFs. These findings indicate that CmARFs have a highly conserved motif in both the DBD and the MR domain.

### Analysis of gene structure and phylogenetic relationships

**Fig. 2:**
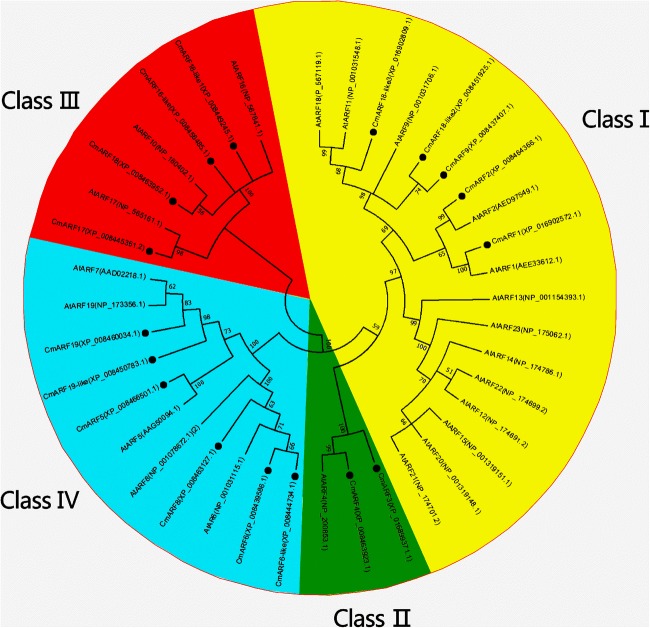
Phylogenetic relationship of putative ARFs in *Arabidopsis thaliana* and melon. The phylogenetic tree was constructed using the NJ method with MEGA 7.0 software. Bootstrap values (1000 replicates) are shown as percentages at the branch nodes. Arabic numerals I–IV represent different ARF groups. The results indicated that ARFs exhibit high homology between melon and *Arabidopsis thaliana*

Phylogenetic analysis involving the deduced amino acid sequences of the 17 melon ARFs and 21 ARFs from *Arabidopsis thaliana* was performed. The results showed that the ARF family genes of the two species could be divided into four subfamilies: classes I, II, III, and IV (Fig. 2). ARFs exhibit high homology between melon and *Arabidopsis thaliana*.

**Fig. 3:**
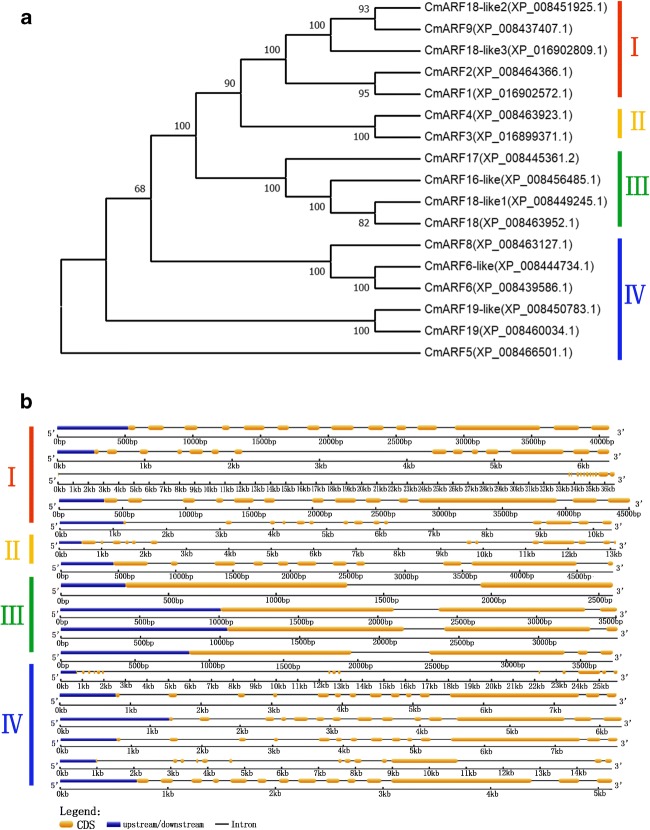
Phylogenetic analysis and intron/exon structures of putative ARF genes in melon. **a**. Phylogenetic analysis of putative ARF genes in melon. ClustalW was used to align the protein sequences of 17 CmARFs. The phylogenetic tree was constructed using the NJ method with MEGA 7.0 software. Bootstrap values (1000 replicates) are shown as percentages at the branch nodes. The Arabic numerals I–IV represent different ARF groups. **b** Intron/exon structures of putative ARF genes in melon. Exons and introns are indicated by open boxes and lines, respectively. The results indicated that ARFs in melon could be divided into four groups: classes I, II, III, and IV

**Fig. 4:**
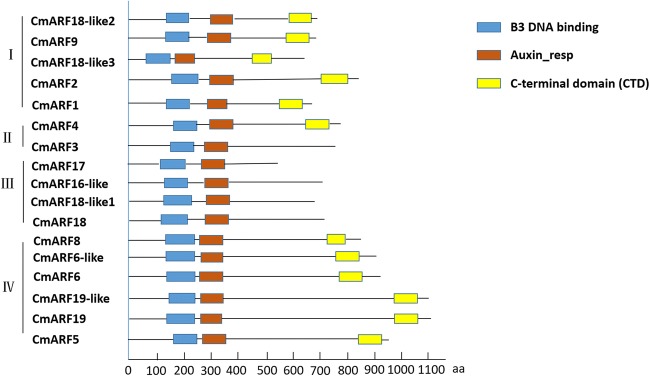
Schematic organization of CmARFs. The auxin_resp domain, B3 DBD, and CTD (Aux/IAA-binding domain) are shown in red, blue, and yellow, respectively. The results indicated that *CmARF16-like*, *CmARF17*, CmARF18, and *CmARF18-like1* contained a DBD and an auxin_resp domain but no CTD

The phylogenetic tree revealed the following information: there are also four subfamilies of ARFs in melon, classes I, II, III, and IV (Fig. 3a). Structural analysis of the identified melon ARF genes revealed differences in the numbers and locations of introns. Most members of the ARF gene family have a complex structure, with approximately 10 introns. There were fewer than five introns in only four ARF genes, namely, *CmARF16-like*, *CmARF17*, *CmARF18*, and *CmARF18-like1* (Fig. 3b)*.* Most CmARFs contained three typical domains: a DBD, an auxin_resp domain, and a CTD. *CmARF3*, *CmARF16-like*, *CmARF17*, *CmARF18*, and *CmARF18-like1* contained a DBD and an auxin_resp domain but no CTD (Fig. 4).

### Tissue-specific expression patterns of *CmARF* genes

**Fig. 5:**
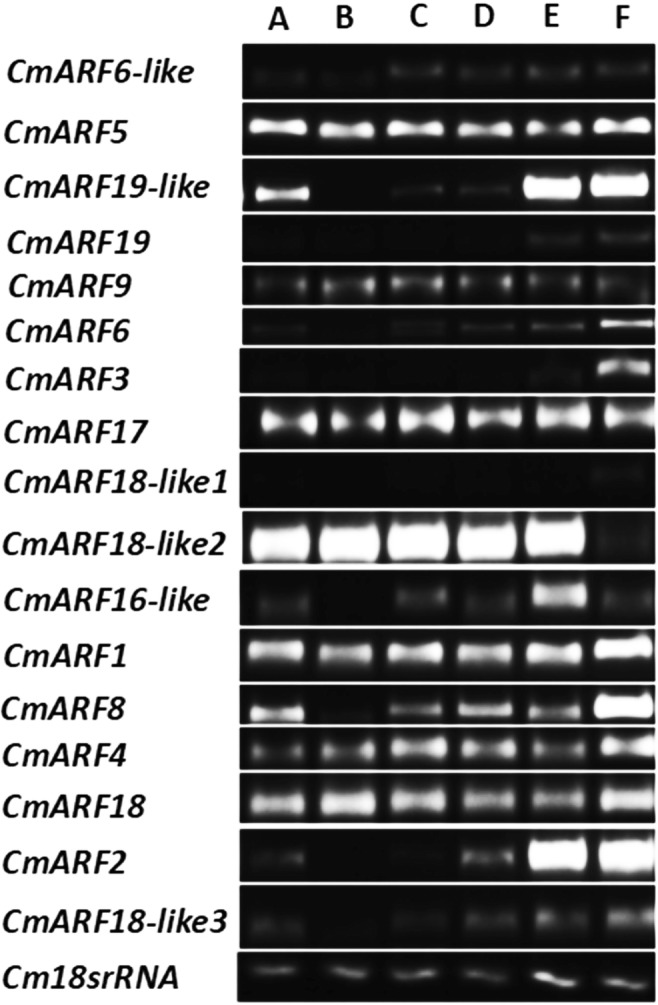
Expression of ARFs in different tissues of melon. Sampling occurred on the day of flowering. (A) Root. (B) Stem. (C) Leaf. (D) Staminate flower petal. (E) Fruit. (F) Pistillate flower petal

To investigate the transcript levels of these CmARFs in different tissues of melon plants, we collected samples of roots, leaves, stems, pistillate flower petals, fruits, and staminate flower petals. Expression analysis via semiquantitative PCR revealed ARF gene expression in those tissues, but the expression levels varied greatly among the different tissues (Fig. 5). *CmARF19*, *CmARF3*, and *CmARF18-like1* were weakly expressed in the young roots; *CmARF19-like*, *CmARF19*, *CmARF6*, *CmARF3*, *CmARF18-like1*, *CmARF16-like*, *CmARF8*, *CmARF2*, and *CmARF18-like3* were barely expressed in the stems; and *CmARF19*, *CmARF3*, and *CmARF18-like1* were barely expressed in the leaves and female flowers. With the exception of *CmARF18-like1*, all the ARF genes were expressed in the fruits. The expression levels of *CmARF2*, *CmARF16-like*, *CmARF18-like2*, and *CmARF19-like* were especially high in the melon fruits, and *CmARF6-like*, *CmARF5*, *CmARF9*, *CmARF17*, *CmARF1*, *CmARF4* and *CmARF18* were expressed in all tissues. The expression levels of *CmARF1*, *CmARF2*, *CmARF3*, *CmARF6*, *CmARF8*, *CmARF18-like3*, and *CmARF19-like* were especially high in the pistillate flower petals.

### Morphological changes in melon fruits during early development

**Fig. 6:**
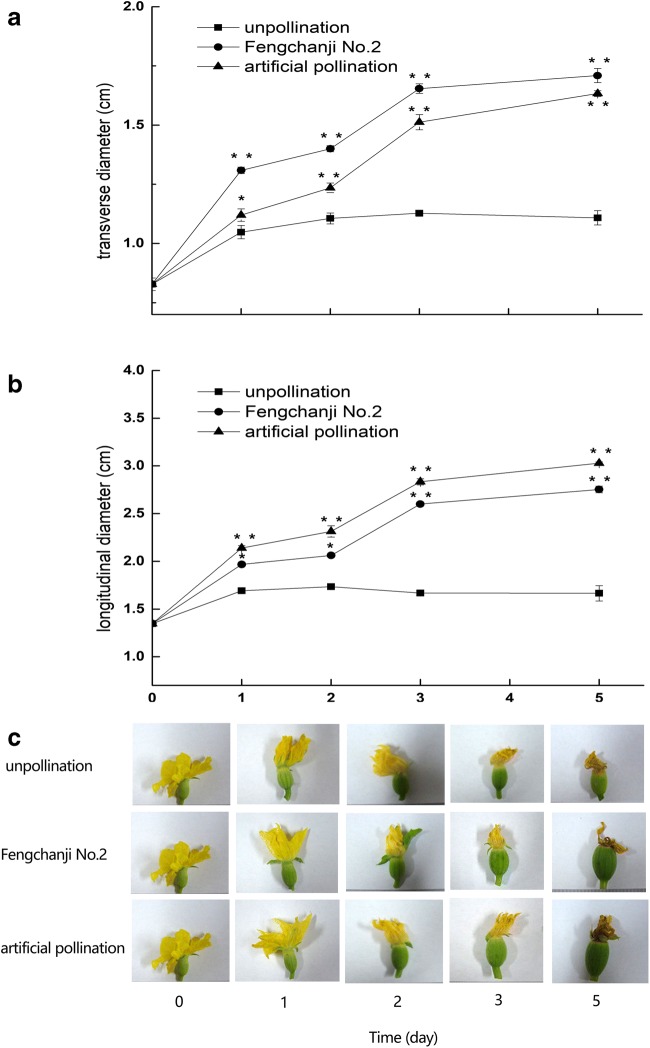
Growth changes on melon fruit after treatment with a growth regulator and artificial pollination. Early development of melon fruit. **a** Transverse diameters of melon fruits during early development. The results indicated that the transverse diameters of the fruits gradually increased after growth regulator treatment. **b** Longitudinal diameters of melon fruits during early development. The results indicated that the longitudinal diameters of the fruits gradually increased after artificial pollination. The data (means ± SDs) are the means of three replicates, with standard errors shown by vertical bars, *n* = 9. * and ** indicate significant differences at *p* < 0.05 and *p* < 0.01, respectively, between the unpollinated group and treatment groups according to Tukey’s test. **c** Shape change in melon fruits during the early development stage. The transverse and longitudinal diameters of the fruits gradually increased after growth regulator treatment and artificial pollination

In the absence of pollination, the fruits exhibited no significant change in either transverse diameter or longitudinal diameter throughout the entire process (Fig. 6a, b). The transverse and longitudinal diameters of the fruits gradually increased after growth regulator treatment and artificial pollination. On the third day of treatment, compared with those in the unpollinated group, the transverse and longitudinal diameters of the fruits in the two treatment groups increased rapidly, resulting in significant differences. During the entire treatment period, compared with those in the unpollinated group, the transverse and longitudinal diameters of the fruits in the growth regulator treatment group increased on average by 38.1% and 25.0%, respectively. Compared with those of the fruits resulting from unpollinated flowers, the average increases in the transverse and longitudinal diameters in the fruits after artificial pollination were 39.1% and 52.9%, respectively (Fig. 6a). The increase in the transverse diameters of the fruits treated with the growth regulator was highly significant, while the increase in the longitudinal diameters of the fruits resulting from artificially pollinated flowers was also highly significant. Images of the fruits during different periods of treatment also demonstrated these findings (Fig. 6c).

### *CmARF* expression during melon fruit development

**Fig. 7:**
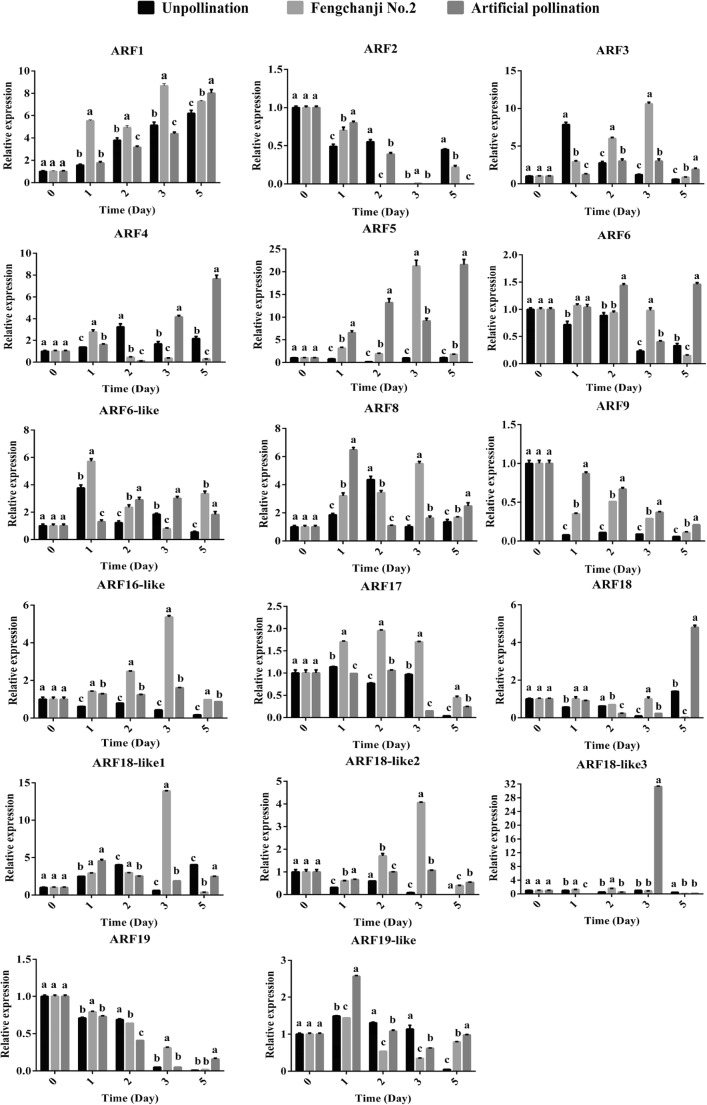
Expression of ARF family genes in melon fruits after growth regulator and artificial pollination treatment. The mRNA expression levels of 17 ARF genes were determined by qRT-PCR. The figure shows the unpollinated group, the artificially pollinated group, and the growth regulator Fengchanji No. 2 treatment group. Fruits were collected at 0, 1, 2, 3, and 5 days after treatment. The data (means ± SDs) are the means of three replicates, with standard errors shown by vertical bars, *n* = 3. The different superscript letters imply a significant difference at *p* < 0.05 between the unpollinated group and the treatment group according to Tukey’s test. The results indicated that different *CmARF* genes exhibit different spatial and temporal expression patterns during the early fruit development of melon

We used qRT-PCR to analyze the expression of ARF genes during the early development of melon fruits after different treatments (Fig. 7). The results showed that, as fruit development progressed, the expression levels of *CmARF1*, *CmARF4*, and *CmARF5* tended to increase in the three treatments, while those of *CmARF2*, *CmARF9*, and *CmARF19* tended to decrease. The expression levels of *CmARF3*, *CmARF6-like*, *CmARF8*, *CmARF16-like*, *CmARF17*, *CmARF18-like1*, *CmARF18-like2*, and *CmARF19-like* tended to increase but then decrease. The trends of the other 3 ARF genes were not obvious.

The expression levels of *CmARF9*, *CmARF16-like*, and *CmARF18-like2* in the two treatment groups were all greater than those in the unpollinated group at the same time points (Fig. 7). In addition, the expression of *CmARF9* in the artificial pollination treatment was greater than that in the growth regulator treatment, and the expression of *CmARF16-like* exhibited the opposite trend. The expression level of *CmARF5* in both treatment groups was greater than that in the unpollinated group. Moreover, the expression level of *CmARF3* in both treatment groups was lower than that in the unpollinated group at first but then was greater than that in the unpollinated group at subsequent time points. The expression of *CmARF1* and *CmARF17* in the growth regulator treatment group was greater than that in the unpollinated group at all stages. The expression of *CmARF1* in the artificial pollination group was first lower than that in the unpollinated group but then was greater than that in the unpollinated group. There was no obvious change in the expression levels of *CmARF17* in the artificial pollination group. Moreover, the expression level of *CmARF4* was lower than that in the unpollinated group beginning on the second day of growth regulator treatment, lower than that in the unpollinated group on the third day of artificial pollination treatment, and greater than that in the unpollinated group for the remainder of the time. The expression level of *CmARF6* was lower than that in the unpollinated group on the 5th day after growth regulator treatment and greater than that in the unpollinated group for the remainder of the time, while the expression in the pollinated group was consistently greater than that in the unpollinated group. The expression level of *CmARF6-like* was lower than that in the unpollinated group on the third day after growth regulator treatment and greater for the remainder of the time. In addition, the expression level of *CmARF6-like* in the artificial pollination group was consistently greater than that in the unpollinated group beginning on the second day. The expression level of *CmARF8* in both treatment groups was lower than that in the unpollinated group on the second day but was greater than that in the unpollinated group for the remainder of the time. Moreover, the expression level of *CmARF18* in the growth regulator treatment group was lower than that in the unpollinated group on the 5th day but greater than that in the unpollinated group for the remainder of the time. The expression level of *CmARF19-like* in the growth regulator treatment group was greater than that in the unpollinated group on the 5th day but lower than that in the unpollinated group for the remainder of the time.

### Correlation analysis between transverse or longitudinal diameter and variation of expression level of *ARF* members

Correlation analysis showed the coefficient between transverse diameter or longitudinal diameter and variation of expression level of *Cm*ARF9, *Cm*ARF19, *Cm*ARF2, and *Cm*ARF1 over 70% in the unpollination group and the two treatment groups (Tables 2 and 3). Moreover, the coefficient between transverse diameter and variation of expression level of *CmARF16-like* and *CmARF3* was significant difference in correlation between unpollination group and the two treatment groups (Table 2), and the coefficient between longitudinal diameter and variation of expression level of *CmARF5*, *CmARF16-like*, and *Cm*ARF19-like was significant difference in correlation between unpollination group and the two treatment groups (Table 3). It suggested that *Cm*ARF9, *CmARF16-like*, *Cm*ARF19-like, *Cm*ARF19, *Cm*ARF1, *Cm*ARF2, *CmARF3*, *and CmARF5* do great related to fruit growth.

**Table 2:** Correlation coefficient (*r*) between transverse diameter and variation of expression level of ARF members

Genes	Unpollination (*r*)	*p* value	Fengchanji no. 2 (*r*)	*p* value	Artificial pollination (*r*)	*p* value
*CmARF1*	0.78	0.04	0.96	0.01	0.92	0.09
*CmARF2*	− 0.85	0.01	− 0.84	0.00	− 0.97	0.01
*CmARF3*	0.09	0.26	0.44	0.15	0.61	0.13
*CmARF4*	0.63	0.06	− 0.37	0.44	0.81	0.26
*CmARF5*	− 0.28	0.19	0.47	0.29	0.85	0.03
*CmARF6*	− 0.69	0.04	− 0.55	0.04	0.01	0.44
*CmARF6-like*	0.11	0.29	0.14	0.20	0.60	0.12
*CmARF8*	0.34	0.20	0.55	0.08	− 0.03	0.25
*CmARF9*	− 0.97	0.00	− 0.95	0.00	− 0.98	0.01
*CmARF16-like*	− 0.74	0.02	0.46	0.33	0.15	0.76
*CmARF17*	− 0.36	0.24	− 0.02	0.95	− 0.85	0.05
*CmARF18*	− 0.30	0.22	− 0.55	0.03	0.51	0.85
*CmARF18-like1*	0.45	0.09	0.42	0.28	0.11	0.08
*CmARF18-like2*	− 0.84	0.01	0.33	0.81	− 0.34	0.05
*CmARF18-like3*	− 0.45	0.12	− 0.41	0.21	0.41	0.41
*CmARF19*	− 0.76	0.03	− 0.92	0.01	− 0.96	0.01
*CmARF19-like*	− 0.10	0.86	− 0.48	0.05	−0.34	0.97

*r* represents correlation coefficient between transverse diameter and variation of expression level of ARF members, *p* value was performed with Pearson’s correlation by *T* test (*p*_0.05_ = 2.776)

**Table 3:** Correlation coefficient (*r*) between longitudinal diameter and variation of expression level of ARF members

Genes	Unpollination(r)	*p* value	Fengchanji no. 2 (*r*)	*p* value	Artificial pollination (*r*)	*p* value
*CmARF1*	0.56	0.09	0.95	0.04	0.88	0.32
*CmARF2*	− 0.71	0.00	− 0.79	0.00	− 0.95	0.00
*CmARF3*	0.36	0.45	0.38	0.28	0.62	0.58
*CmARF4*	0.67	0.51	− 0.42	0.06	0.76	0.69
*CmARF5*	− 0.48	0.00	0.48	0.37	0.84	0.05
*CmARF6*	− 0.46	0.00	− 0.63	0.00	0.01	0.01
*CmARF6-like*	0.31	0.92	0.08	0.61	0.62	0.54
*CmARF8*	0.52	0.65	0.47	0.35	0.05	0.85
*CmARF9*	− 0.98	0.00	− 0.93	0.00	− 0.95	0.00
*CmARF16-like*	− 0.57	0.00	0.43	0.91	0.21	0.01
*CmARF17*	− 0.22	0.00	− 0.16	0.07	− 0.80	0.00
*CmARF18*	− 0.32	0.00	− 0.60	0.00	0.45	0.35
*CmARF18-like1*	0.56	0.30	0.40	0.43	0.22	0.81
*CmARF18-like2*	− 0.71	0.00	0.31	0.43	− 0.35	0.00
*CmARF18-like3*	− 0.44	0.00	− 0.54	0.01	0.41	0.51
*CmARF19*	− 0.52	0.00	− 0.96	0.00	− 0.96	0.00
*CmARF19-like*	0.10	0.04	− 0.49	0.00	− 0.25	0.04

*r* represents correlation coefficient between longitudinal diameter and variation of expression level of ARF members, *p* value was performed with Pearson’s correlation by *T* test (*p*_0.05_ = 2.776)

## Discussion

Auxin is associated with all stages of plant growth and development, such as cell division, cell elongation and differentiation, apical dominance, plant orientation, senescence, shedding, and, in certain plant species, the induction of blooming (Marc et al. 2007). Auxin regulates these processes by controlling gene expression via a series of uniquely functional DNA-binding ARFs (Mcphie 2002). In recent years, given the complete genome sequencing of multiple species, studies on the plant ARF family have been carried out gradually for different species. The identification of the ARF family began with *Arabidopsis* (Tamura et al. 2011), and the identification of ARF family members is the basis for studying the regulatory mechanism and function of ARFs in plant growth and development.

In this study, we identified 17 ARF genes in melon. There are fewer *CmARF* members in melon than in both *Arabidopsis* (23) and rice (25) (Tamura et al. 2011; Goff 2002). The genome size of melon is approximately the same as that of rice but is very different from that of *Arabidopsis* (melon: 450 Mb, *Arabidopsis*: 125 Mb, rice: 420 Mb) (Weng et al. 2017; Livak and Schmittgen 2001). Thus, the number of ARF genes in species with large genomes is not always large. Most members of the CmARF gene family have a complex structure with approximately 10 introns. Subcellular localization predicted that all of these genes except *CmARF15* were localized in the nucleus. We also constructed a phylogenetic tree to analyze the relationships among ARF family genes in melon. In addition, *CmARF8* and *AtARF8* were highly homologous, reaching 96%. *CmARF16-like*, *CmARF17*, *CmARF18*, and *CmARF18-like1* in melon contain a DBD and an auxin_resp domain but not a CTD (Aux/IAA-binding domain). Other CmARFs contain three typical domains: a DBD, an auxin_resp domain, and a CTD.

ARFs exhibit tissue-specific expression. In *Arabidopsis*, *ARF9* is expressed in all tissues (Philip et al. 2004). In addition, *GrARF1*, *GrARF2a*, *GrARF3a*, *GrARF4a*, *GrARF5a*, *GrARF6b*, *GrARF16c*, *GrARF17a*, *GrARF19.1a*, and *GrARF19.2* are highly expressed in flower buds of *Gossypium raimondii*, and *GrARF1*, *GrARF4a*, *GrARF5a*, *GrARF16c*, *GrARF17a*, and *GrARF19.1a* are highly expressed in the leaves (Sun et al. 2015). We performed semiquantitative PCR to analyze the differences in expression among different tissues during the early development of melon. We found that the expression levels of *CmARF2*, *CmARF16-like*, *CmARF18-like2*, and *CmARF19-like* were especially high in melon fruits. Moreover, the expression levels of *CmARF1*, *CmARF2*, *CmARF3*, *CmARF6*, *CmARF8*, *CmARF18-like3*, and *CmARF19-like* were especially high in pistillate flower petals; and *CmARF1*, *CmARF4*, *CmARF5*, *CmARF6-like*, *CmARF9*, *CmARF17*, and *CmARF18* were expressed in all tissues. Thus, the expression of ARFs in melon is tissue specific. Studies have shown that *ARF2* regulates the formation of floral organs (Finet et al. 2010) and that *ARF5* is associated with embryonic development and vascular bundle formation (Weijers et al. 2006; Wenzel et al. 2010). The homologous genes of *AtARF2* and *AtARF5* in melon, *CmARF2*, and *CmARF5*, were identified. *CmARF2* is highly expressed in fruits and female flowers, while *CmARF5* is highly expressed in all parts. Therefore, we speculate that ARFs whose expression differs in different tissues have different functions during plant growth and development.

ARFs participate in the transcriptional regulation of fruit development (Johnson et al. 2007; Bohner and Bangerth 1988; De et al. 2008). In this study, the fruits resulting from the pollinated flowers quickly expanded, while the unpollinated ovaries did not grow. *CmARF9*, *CmARF16-like*, *CmARF19-like*, *CmARF19*, *CmARF1*, *CmARF2*, *CmARF3*, and *CmARF5* may be associated with the fruit growth of melon during early development. In tomato, *ARF9* negatively controls cell division during early fruit development. The transcription level of *SlARF9* increased within 48 h after pollination but subsequently decreased in the following days (Wang et al. 2005). The high expression of *CmARF9* before pollination decreased after artificial pollination or growth regulator treatment in this study, similar to that of *SlARF9* in tomato. ARF19 is induced by either IAA or ethylene treatments, not only participates in auxin signaling, but also plays a critical role in ethylene responses in Arabidopsis (Jisheng Li et al. 2019). Decreased expression of *CmARF9* may be related to cell division and early fruit development. It is worth noting that in the unpollinated ovaries, *ARF9* expression decreased sharply 1 day after flowering, which was significantly faster than that in the treatment group. We hypothesized that *ARF9*, in addition to promoting the regulation of cell division to regulate fruit enlargement, was also involved in other biochemical reactions in plants. *ARF16* has been shown to be involved in the regulation of root crown cell differentiation in *Arabidopsis* (Wang et al. 2005). *ARF3* regulates floral meristem determinacy by repressing cytokinin biosynthesis and signaling (Zhang et al. 2018). *ARF3* also regulates compound leaf patterns in *Medicago truncatula* (Peng et al. 2017), and *ARF5* has been shown to mediate root meristem formation in *Arabidopsis* and poplar (Berleth and Jurgens 1993; Johnson and Douglas 2007). However, relationships between *ARF16-like*, *ARF3*, and *ARF5* and fruit development have not been reported, which warrants further discussion in the future. It is interesting that the transverse diameter of the fruit obviously increased after growth regulator treatment, while the longitudinal diameter of the fruit obviously increased after artificial pollination. By analyzing the expression of ARFs during fruit development of melon, we found that the expression of *CmARF9* in the artificial pollination treatment was greater than that in the growth regulator treatment, and *CmARF16-like* exhibited the opposite trend. The expression of *CmARF3* in the growth regulator treatment was greater than that in the artificial pollination treatment after 2 days, and the opposite trend was observed on day 5 after treatment. Based on the changes in ARF expression and fruit growth in each group, it was suggested that *CmARF16-like* and *CmARF3* were correlated with the horizontal cell growth of melon fruit, while *CmARF9* was correlated with the vertical cell growth of melon fruit. The duration, rate, and direction of cell division in developing ovaries may have significantly affected the final fruit size and shape (Bohner and Bangerth 1988). Hence, the relationship of the mechanism between ARFs and the transverse or longitudinal diameter needs further study.

*SlARF8* and *SlARF7* in tomato are involved in auxin signal transduction during fruit set and development (De et al. 2008). *ARF8* transcription in *Arabidopsis thaliana* and tomato increases during floral development, and the expression level remains high in unpollinated ovaries but decreases after pollination (Marc et al. 2007). In this study, the expression level of *CmARF8* in the pollinated group and the growth regulator treatment group was lower than that in the unpollinated group on the second day after pollination but greater than that in the unpollinated group during the rest of the time. Before pollination, the level of *CmARF8* expression was low, but it increased after pollination or after growth regulator treatment. These findings contrast with results in tomato (Marc et al. 2007), possibly because *ARF8* functions differently in different species.

## Conclusion

In summary, we proposed a model to explain this phenomenon. When the ovary was not pollinated, Aux/IAA suppressors formed homologous dimers with ARFs; consequently, ARF activity was inhibited, and auxin signal transduction was inhibited, resulting in fruit failure. After ovary pollination, auxin increases rapidly, Aux/IAA inhibitors are degraded by the ubiquitination pathway, ARFs are released, auxin response genes are activated, and then fruit set or fruit development is initiated. Verification of this prediction and investigation of other possible modes of action of ARFs will constitute our future research goals.

## Abbreviations

ARF: Auxin response factor
Aux/IAA: Auxin/indole-3-acetic acid
GH3: Gretchen Hagen 3
SAUR: Small auxin-up RNA
AuxRE-core: Core element of the auxin response region
AuxRE: Auxin response element
DBD: DNA-Binding domain
CTD: C-Terminal dimerization domain
MR: Middle region
AD: Activation dimer
RD: Repression dimer
qRT-PCR: Quantitative real-time PCR

## References

[CR1] Bailey TL, Boden M, Buske FA, Frith M, Grant CE, Clementi L, Ren J, Li WW, Noble WS (2009). MEME SUITE: tools for motif discovery and searching. Nucleic Acids Res.

[CR2] Berleth T, Jurgens G (1993). The role of the monopteros gene in organising the basalbody region of the Arabidopsis embryo. Development.

[CR3] Bohner J, Bangerth F (1988). Effects of fruit set sequence and defoliation on cell number, cell size and hormone levels of tomato fruits (*Lycopersicon esculentum*mill.) within a truss. Plant Growth Regul.

[CR4] Davies PJ (1987) The plant hormones: their nature, occurrence, and functions. Plant Horm Physiol Biochem Mol Biol:1–2. 10.1007/978-94-011-0473-9_1

[CR5] De JM, Wolters-Arts MR, Mariani C, Vriezen WH (2008). The solanum lycopersicum auxin response factor 7 (SlARF7) regulates auxin signaling during tomato fruit set and development. Plant.

[CR6] Dolf W, Eva B, Ger KE, Alexandra S, Thorsten H, Marika K, Wilmoth JC, Reed JW, Gerd J (2014). Developmental specificity of auxin response by pairs of arf and aux/iaa transcriptional regulators. EMBO J.

[CR7] Ellis CM, Punita N, Young JC, Gretchen H, Guilfoyle TJ, Reed JW (2005) *Auxinresponsefactor1* and *auxinresponsefactor2* regulate senescence and floral organ abscission in Arabidopsis thaliana. Development:132, 4563–4574. 10.1242/dev.02012 10.1242/dev.02012 16176952

[CR8] ElsharkawyI SSM, Jones B, Mila I, Kumar PP, Bouzayen M, Jayasankar S (2014). Tir1-like auxin-receptors are involved in the regulation of plum fruit development. J Exp Bot.

[CR9] Finet C, Fourquin C, Vinauger M, Berne-Dedieu A, Chambrier P, Paindavoine S, Scutt CP (2010). Parallel structural evolution of auxin response factors in the Tngiosperms. Plant.

[CR10] Garcia-Mas J, Benjak A, Sanseverino W, Bourgeois M, Mir G, González VM, Hénaff E, Câmara F, Cozzuto L, Lowy E, Alioto T, Capella-Gutiérrez S, Blanca J, Cañizares J, Ziarsolo P, Gonzalez-Ibeas D, Rodríguez-Moreno L, Droege M, Du L, Alvarez-Tejado M, Lorente-Galdos B, Melé M, Yang L, Weng Y, Navarro A, Marques-Bonet T, Aranda MA, Nuez F, Picó B, Gabaldón T, Roma G, Guigó R, Casacuberta JM, Arús P, Puigdomènech P (2012). The genome of melon (*Cucumis melo* L.). Proc Natl Acad Sci.

[CR11] Goetz M, Hooper LC, Johnson SD, Rodrigues JCM, Viviansmith A, Koltunow AM (2007). Expression of aberrant forms of stimulates parthenocarpy in Arabidopsis and tomato. Plant Physiol.

[CR12] Goff SA (2002). A draft sequence of the rice genome (*Oryza sativa*L. *ssp. japonica*). Science.

[CR13] Guilfoyle TJ, Hagen G (2007). Auxin response factors. Curr Opin Plant Biol.

[CR14] Guo AY, Zhu QH, Xin C (2007). Gsds:a gene structure display server. Hereditas.

[CR15] Li J, Dai X, Zhao Y (2019). A role for auxin response factor 19 in auxin and ethylene signaling in Arabidopsis. Plant Physiol.

[CR16] Johnson LA, Douglas CJ (2007). Populus trichocarpa monopteros/auxinresoonsefactor5 (ARF5) genes: comparative structure, sub-functionalization, and populus-arabidopsis microsynteny. Can J Bot.

[CR17] Lim PO, Lee IC, Kim J, Kim HJ, Ryu JS, Woo HR, Hong GN (2010). Auxin response factor 2 (ARF2) plays a major role in regulating auxin-mediated leaf longevity. J Exp Bot.

[CR18] Liu Y, Jiang HY, Chen WJ, Qian YX, Ma Q, Cheng BJ, Zhu SW (2011). Genome-wide analysis of the auxin response factor (ARF) gene family in maize (Zea mays). Plant Growth Regul.

[CR19] Livak KJ, Schmittgen TD (2001). Analysis of relative gene expression data using real-time quantitative PCR and the 2^−ΔΔCT^ method. Methods.

[CR20] Marc G, Adam VS, Johnson SD, Koltunow AM (2006). Auxinresponsefactor8 is a negative regulator of fruit initiation in Arabidopsis. Plant Cell.

[CR21] Marc G, Hooper LC, Johnson SD, Rodrigues JCM, Adam VS, Koltunow AM (2007). Expression of aberrant forms of auxinresponsefactor8 stimulates parthenocarpy in Arabidopsis and tomato. Plant Physiol.

[CR22] Mcphie P (2002). The protein protocols handbook. Anal Biochem.

[CR23] Park JE, Park JY, Kim YS, Staswick PE, Jeon J, Yun J, Kim SY, Kim J, Lee YH, Park CM (2007). Gh3-mediated auxin homeostasis links growth regulation with stress adaptation response in *Arabidopsis*. J Biol Chem.

[CR24] Peng J, Berbel A, Madueño F, Chen R (2017) Auxinresponsefactor3 regulates compound leaf patterning by directly repressing palmate-likepentafoliat1 expression in Tedicago truncatula. Front Plant Sci, 8:1630. 10.3389/fpls.2017.01630 10.3389/fpls.2017.01630 PMC5611443 28979286

[CR25] Philip Z, Matthias HH, Lars H, Wilhelm G (2004). Genevestigator. Arabidopsis microarray database and analysis toolbox. Plant Physiol.

[CR26] Piya S, Shrestha SK, Binder B, Stewart CN Jr, Hewezi T (2014) Protein-protein interaction and gene co-expression maps of ARFs and aux/IAAs in Arabidopsis. Front Plant Sci 5. 10.3389/fpls.2014.00744 10.3389/fpls.2014.00744 PMC4274898 25566309

[CR27] Shen C, Yue R, Sun T, Zhang L, Cu LQ, Tie SG, Wang HZ, Yang YJ (2015). Genome-wide identification and expression analysis of auxin response factor gene family in Medicago truncatula. Front Plant Sci.

[CR28] Sun R, Wang K, Guo T, Jones DC, Cobb J, Zhang B, Wang Q (2015). Genome-wide identification of auxin response factor (ARF) genes and its tissue-specific prominent expression in Tossypium raimondii. Funct Integr Genomics.

[CR29] Tamura K, Peterson D, Peterson N, Stecher G, Nei M, Kumar S (2011) Mega5: molecular evolutionary genetics analysis using maximum likelihood, evolutionary distance, and maximum parsimony methods. Mo Biol Evol 28:2731-2739.10.1093/molbev/msr121 10.1093/molbev/msr121 PMC3203626 21546353

[CR30] Tatematsu K, Kumagai S, Muto H, Sato A, Watahiki MK, Harper RM, LiscumE YKT (2004). Massugu2 encodes aux/iaa19, an auxin-regulated protein that functions together with the transcriptional activator nph4/arf7 to regulate differential growth responses of hypocotyl and formation of lateral roots in *Arabidopsis thaliana*. Plant Cell.

[CR31] Thorsten H, Eva B, Isabel BU, Marika K, Gerd J (2002). The Arabidopsis bodenlos gene encodes an auxin response protein inhibiting monopteros-mediated embryo patterning. Genes Dev.

[CR32] Tiwari SB, Gretchen H, Tom G (2003). The roles of auxin response factor domains in auxin-responsive transcription. Plant Cell.

[CR33] Tiwari SB, Wang XJ, Hagen G, Guilfoyle TJ (2001). Aux/iaaproteins are active repressors, and their stability and activity are modulated by auxin. Plant Cell.

[CR34] Ulmasov T, Hagen G, Guilfoyle TJ (1999). Activation and repression of transcription by auxin-response factors. Proc Natl Acad Sci U S A.

[CR35] Vanneste S, Friml J (2009). Auxin: atrigger for change in plant development. Cell.

[CR36] Wang JW, Wang LJ, Mao YB, Cai WJ, Xue HW, Chen XY (2005). Control of root cap formation by microRNA-targeted auxin response factors in Arabidopsis. Plant Cell.

[CR37] Weijers D, Schlereth A, Ehrismann JS, Schwank G, Kientz M, Jürgens G (2006). Auxin triggers transient local signaling for cell specification in Arabidopsis embryogenesis. Dev Cell.

[CR38] Weng Q, Song J, Ma H, Yuan J, Liu Y (2017) Cloning and expression analysis of zmabi3 gene in Zea mays. Turk J Biochen 42. 10.1515/tjb-2016-0303

[CR39] Wenzel CL, Mathias S, Qian Y, Jim M (2010). Dynamics of monopteros pteros and pin-rormed1 expression during leaf vein pattern formation in Arabidopsis thaliana. Plant J.

[CR40] Wilmoth JC, Shucai W, Tiwari SB, Joshi AD, Gretchen H, Guilfoyle TJ, Alonso JM, Ecker JR, Reed JW (2010). Nph4/arf7 and arf19 promote leaf expansion and auxin-induced lateral root formation. Plant J Cell Mol Biol.

[CR41] Yang C, Xu M, Xuan L, Jiang X, Huang M (2014). Identification and expression analysis of twenty ARF genes in Populus[J]. Gene.

[CR42] Yoko O, Hidehiro F, Makoto O, Athanasios T, Masao T (2007). arf7 and arf19 regulate lateral root formation via direct activation of lbd/asl genes in Arabidopsis. Plant Cell.

[CR43] Zhang K, Wang R, Zi H, Li Y, Cao X, Li D, Guo L, Tong J, Pan Y, Jiao Y (2018) Auxinrespovsefactor3 regulates floral meristem determinacy by repressing cytokinin biosynthesis and signaling. Plant Cell 30. 10.1105/tpc.17.00705 10.1105/tpc.17.00705 PMC5868698 29371438

[CR44] Zhang S, Chen S, Chen F, Fang W (2012). The regulatory role of the auxin in the creeping chrysanthemum habit. Russ J Plant Physiol.

